# *Lactobacillus rhamnosus* GG Regulates Host IFN-I Through the RIG-I Signalling Pathway to Inhibit Herpes Simplex Virus Type 2 Infection

**DOI:** 10.1007/s12602-023-10137-8

**Published:** 2023-08-25

**Authors:** Jingyu Wang, Mei Huang, Yuqi Du, Haoming Chen, Zixiong Li, Taiyu Zhai, Zihao Ou, Yiyi Huang, Fan Bu, Haojun Zhen, Ruoru Pan, Yubing Wang, Xiaohan Zhao, Bo Situ, Lei Zheng, Xiumei Hu

**Affiliations:** 1 Department of Laboratory Medicine, Nanfang Hospital, Southern Medical University, Guangzhou, China; 2 Center for Clinical Laboratory, Zhujiang Hospital, Southern Medical University, Guangzhou, China

**Keywords:** Commensal microbiota, Herpes simplex virus type 2, RIG-I, Interferon type I, *Lactobacillus rhamnosus* GG

## Abstract

Numerous recent studies have demonstrated that the commensal microbiota plays an important role in host immunity against infections. During the infection process, viruses can exhibit substantial and close interactions with the commensal microbiota. However, the associated mechanism remains largely unknown. Therefore, in this study, we explored the specific mechanisms by which the commensal microbiota modulates host immunity against viral infections. We found that the expression levels of type I interferon (IFN-I) and antiviral priming were significantly downregulated following the depletion of the commensal microbiota due to treatment with broad-spectrum antibiotics (ABX). In addition, we confirmed a unique molecular mechanism underlying the induction of IFN-I mediated by the commensal microbiota. In vivo and in vitro experiments confirmed that *Lactobacillus rhamnosus* GG (LGG) can suppress herpes simplex virus type 2 (HSV-2) infection by inducing IFN-I expression via the retinoic acid-inducible gene-I (RIG-I) signalling pathway. Therefore, the commensal microbiota-induced production of IFN-I provides a potential therapeutic approach to combat viral infections. Altogether, understanding the complexity and the molecular aspects linking the commensal microbiota to health will help provide the basis for novel therapies already being developed.

**Graphical Abstract:**

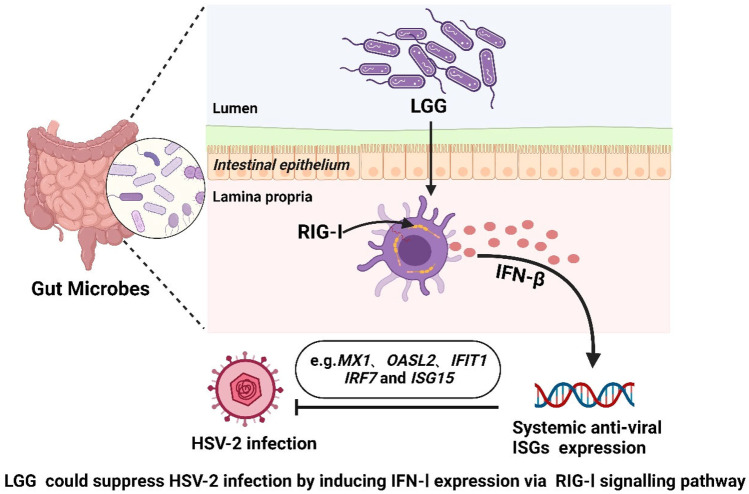

**Supplementary Information:**

The online version contains supplementary material available at 10.1007/s12602-023-10137-8.

## Introduction

Herpes simplex virus type 2 (HSV-2), the most prevalent and widespread sexually transmitted pathogen globally, primarily causes genital herpes by infecting the skin and mucous membranes of the lower genital tract [1–3]. This virus can also remain dormant in the ganglia, causing recurrent infections [4, 5]. Additionally, HSV-2 infection is closely associated with the pathogenesis of reproductive tract malignancies, such as cervical cancer, penile cancer and prostate cancer; it has increased their incidences [6–8]. HSV-2 infection has also significantly increased the risk of human immunodeficiency virus 1 (HIV-1) infection owing to its epidemiological synergy with HIV-1 infection and transmission [3, 9, 10]. Recently, there has been a rapid increase in the global incidence and recurrence rates of HSV-2 infection, but an effective drug to treat this infection is still lacking [1, 2]. The main challenge associated with this is the ability of this virus to establish recurrent infections and its lifelong latency, as it can suppress the host immune response and evade immune recognition by maintaining latency in the dorsal root ganglion [11]. Hence, it is of scientific and clinical significance to search for an effective therapeutic method that functions by targeting the mechanisms underlying viral pathogenicity in the host.

Interferons (IFNs), as host-produced antiviral compounds, play a pivotal role in resisting viral infections [12–14]. Among them, type I IFN (IFN-I) have been employed for treating various viral infections for more than 60 years owing to their potent antiviral effects [15]. IFN-I, which includes IFN-α and IFN-β, can be secreted by cells in an autocrine and paracrine manner to activate a signalling cascade. By binding to the IFN-I receptor (IFNAR), IFN-I signals to the nucleus, triggering the expression of a large number of IFN-stimulated genes (ISGs) with antiviral activities [16–18]. In fact, by establishing a restrictive antiviral state in cells and regulating immune cell subsets essential for the antiviral response, IFN-I is a major player in the treatment of most viral infections [19–21]. Given the importance of IFN-I for organismal health, it is necessary to better understand mechanistically how the constitutive IFN-I immune response is regulated in normal individuals and subsequently dysregulated, giving rise to disease.

Our bodies are colonised by trillions of microorganisms, collectively known as the commensal microbiota [22, 23]. The commensal microbiota passively colonise and exert antiviral effects by modulating the maturation of immune cells and inducing the release of immune factors [24–26]. However, how the human microbiome influences viral infections remains a mystery. The composition of the commensal microbiota in hamsters changed significantly following SARS-CoV-2 infection in a manner correlated with the severity of the infection [27]. Probiotics have also been implicated in inhibiting viral respiratory infections by blocking the binding of viruses to the target host cells and modulating the anti-virus natural immune response [28].

Further, in a mouse model of influenza infection, it was revealed that administering *Lactobacillus rhamnosus* GG (LGG), which is an important intestinal probiotic, can reduce the viral load and inhibit viral infection by activating autoimmune responses [29, 30]. These results confirm that *lactobacilli* can regulate viral infections, but the regulatory mechanisms underlying the effects of *lactobacilli* on viral infections of the female reproductive and genital tract remain unclear. Further, previous studies mostly involved *Lactobacillus* spp., which has been shown to be important in the defence of the female genital tract against a wide range of urogenital pathogens, including HIV-1 [31, 32]. As a microecological regulator of reproductive vaginal viral infections, LGG is one of the most widely adopted probiotics [33–35]. Therefore, we hypothesised that LGG protects against viral infection by modulating the host immune system. We explored the specific mechanisms by which the commensal microbiota modulates host immunity against such viral infections. In this study, we demonstrate that the commensal microbiota can induce systemic tonic IFN-I and antiviral priming. Moreover, we identified a specific molecular mechanism underlying the commensal microbiota-mediated induction of IFN-I. In vitro and in vivo experimentation results suggest that LGG can suppress HSV-2 infection by inducing IFN-I expression via the retinoic acid-inducible gene-I (RIG-I) signalling pathway.

## Materials and Methods

### Bacterial Strains and Bacterial Growth Condition

In this experiment, LGG (ATCC 53103) was used, which is a strain of *Lactobacillus* and a gram-positive facultative anaerobe. The strain was inoculated into MRS Liquid medium (027312, HuanKai Microbial, Guangdong, China) at 37 °C, 160 rpm, cultured in an anaerobic environment with GENbag (45534 Biomerieux, French) shaken for 24 h, and then centrifuged at 5000 × g for 5 min and resuspended in PBS to obtain concentration of 10^8^ CFU/mL.

### Broad-Spectrum Antibiotics (ABX) Treatment

To establish the gut microbiota-depleted specific pathogen free (SPF) mouse model, after 1 week of acclimation, ABX treatment was performed according to the following protocol. The broad-spectrum antibiotic cocktail consisted of four antibiotics: neomycin (1 g/L), ampicillin (1 g/L), metronidazole (1 g/L) and vancomycin (0.5 g/L). The mixture was dissolved in sterilised water. Mice received the ABX solution by intragastric gavage (i.g). for at least 10 days at 200 μL per mouse per day. After 10 days i.g, faecal samples were collected and aerobically and anaerobically cultured to determine microbiota depletion. We used qPCR to quantify of bacterial 16S rDNA in faeces of mice treated (or not) with ABX. During the treatment period, the weight changes and physical states of the mice were monitored daily. The antibiotics were replaced with regular sterilised water after stopping the ABX treatment to enable microbiota recolonisation.

### Animal Experiments

Regular SPF mice were fed under SPF conditions in the Southern Medical School experimental animal centre. All wild type (WT) and IFNAR1-knockout (Ifnαr1−/−) mice were experimented in the adult stage (typically 6–8 weeks) and only females were used in this study. Two types of mice were used in this experiment: WT SPF mice and Ifnαr1^−/−^ SPF mice. The mice were administered PBS (200 μL each mouse) or LGG (10^8^ CFU/mL, 200 μL each mouse) by i.g. every other day for 14 days, and then their organs were removed to extract RNA for ISGs expression analysis. Experiments in vivo were performed in technical triplication and were repeated three times independently. All animal experimentation was approved by the Southern Medical University Institutional Animal Care and Use Committee (IACUC) (Approval protocol no. SMUL2021045).

### Quantitative Reverse Transcription Polymerase Chain Reaction (qRT-PCR) Gene Expression Analysis

After 10 days of ABX treatment, RNA was extracted from the thymus, spleen, mLN and colon of WT and Ifnαr1^−/−^ female SPF mice using the EasyPure® RNA Purification Kit (ER701-01, TransGen Biotech, Beijing, China) and converted into complementary DNA using the *EasyScript*® All-in-One First-Strand cDNA Synthesis SuperMix (AE341-02, TransGen Biotech) for qRT-PCR, which was performed with the Automatic Fluorescence Quantitative PCR instrument (LightCycler®480 II, Roche) using TransStart® Top Green qPCR SuperMix (+Dye I) kit (AQ132-11, TransGen Biotech). To standardise the expression level of each gene, gene expression was normalised by GAPDH. All the primers used in the experiment were showed in Table 1.

**Table 1:** List of primers

**Gene**	**F/R**	**Mouse**	**Human**
GAPDH	F	AGAACATCATCCCTGCCTCTACT	CACCCACTCCTCCACCTTT
	R	GATGTCATCATATTTGGCAGGTT	CTTCCTCTTGTGCTCTTGC
IFNβ	F	CAGCTCCAAGAAAGGACGAAC	GCTTGGATTCCTACAAAGAAGCA
	R	GGCAGTGTAACTCTTCTGCAT	ATAGATGGTCAATGCGGCGTC
ISG15	F	GGTGTCCGTGACTAACTCCAT	AGGACAGGGTCCCCCTTGC
	R	TGGAAAGGGTAAGACCGTCCT	CCGCTCACTTGCTGCTTCA
IRF7	F	GAGACTGGCTATTGGGGGAG	GCTGGACGTGACCATCATGTA
	R	GACCGAAATGCTTCCAGGG	GGGCCGTATAGGAACGTGC
OASL2	F	TTGTGCGGAGGATCAGGTACT	CCATTGTGCCTGCCTACAGAG
	R	TGATGGTGTCGCAGTCTTTGA	CTTCAGCTTAGTTGGCCGATG
MX1	F	GACCATAGGGGTCTTGACCAA	GGTGGTCCCCAGTAATGTGG
	R	AGACTTGCTCTTTCTGAAAAGCC	CGTCAAGATTCCGATGGTCCT
IFIT1	F	CTGAGATGTCACTTCACATGGAA	AGAAGCAGGCAATCACAGAAAA
	R	GTGCATCCCCAATGGGTTCT	CTGAAACCGACCATAGTGGAAAT
DDx58	F	GAG AGT CAC GGG ACC CAC T	GCCATTACACTGTGCTTGGAGA
	R	CGG TCT TAG CAT CTC CAA CG	CCAGTTGCAATATCCTCCACCA
MDA5	F	TGATGCACTATTCCAAGAACTAAC	GAGCAACTTCTTTCAACCACAG
	R	TCTGTGAGACGAGTTAGCCAAG	CACTTCCTTCTGCCAAACTTG
HSV-2 gG	F	CCCACACCCCAACACATC	
	R	CCAAGGCGACCAGACAAAC	

### Cell Culture and Infection

HEK293T cells (GNHu17, Shanghai Institute of Biochemistry and Cell Biology, China) were cultured in 90% Dulbecco’s modified Eagle’s medium (12800017, Gibco, USA) with 10% foetal bovine serum (10099, Gibco, USA), at 37 °C with 5% CO_2_ cell culture chamber (MCO-170ACL-PC, PHCbi, Japan). To explore whether the RIG-I signalling pathways regulate the ISGs expression in vitro, HEK293T cells were treated with RIG-I inhibitor (Cyclo Phe-Pro, 100 μM) (HY-P1934, Med Chem Express, China), RIG-I agonists (KIN1408, 10 μM) (HY-19961, Med Chem Express, China) or DMSO (0.1%) and incubated for 12 h before adding IFN-β to stimulate the cells for 2 h. Then the cells were harvested to isolate RNA for ISGs expression analysis via qRT-PCR. To investigate whether the RIG-I signalling pathways influence the HSV-2 infection in vitro, HEK293T cells were treated with RIG-I inhibitor (Cyclo Phe-Pro, 100 μM), RIG-I agonists (KIN1408, 10 μM), DMSO (0.1%) or acyclovir (6 μM) (59277-89-3, Meilunbio, China) which was to be the positive control and incubated for 12 h before HSV-2 infection. Then, we observed the cytopathic effect under confocal fluorescence microscopy and analysed the viral titre in the culture supernatant of HSV-2-infected HEK293T cells using qRT-PCR.

### Immunofluorescence

HEK293T cells were treated with RIG-I inhibitor (Cyclo Phe-Pro, 100 μM), RIG-I agonists (KIN1408, 10 μM), DMSO (0.1%) or acyclovir (6 μM) and incubated for 12 h before HSV-2 infection. Then, the cells were fixed by 4% paraformaldehyde in confocal dishes, followed by lipid bilayer cell membrane breaking using 0.3% TritonX-100, 5% BSA for closure, followed by incubation with mouse monoclonal antibody to HSV1 + HSV2 gB (ab6506, Abcam, UK), followed by staining treatment with goat anti-mouse FITC secondary antibody (SSA020, Sino Biological, China) and DAPI (C1005, Beyotime, China). All mentioned above steps were performed after three times washing with PBS. After the pre-processing, confocal fluorescence microscopy was performed with a 40 × objective, and fluorescence was selected at FITC (488 nm) and DAPI (405 nm).

### Mouse HSV-2 Infection

To establish the HSV-2 infection model, C57BL/6 female mice were injected subcutaneously in the thigh with 2 mg per mouse of sterile PBS suspension of medroxyprogesterone acetate (XianJu Pharmaceutical Co., Ltd., Taizhou, China) on day 7 prior to infection. On the seventh day after the medroxyprogesterone acetate injection, C57BL/6 female mice were anaesthetised via the intraperitoneal injection of 2% pentobarbital sodium. Then, the mice were attacked with HSV-2 via vaginal instillation, using 10 μL each time, for a total of 20 μL per mouse, making the attack doses to be 1.92 × 10^7^ PFU/mL. The body weight, clinical signs and symptoms and survival of the mice were monitored on a daily basis. Based on previous studies, the disease symptoms of HSV-2 challenged mice were scored as follows: grade 0–5 mean healthy; genital erythema; moderate genital inflammation; genital lesion; hind limb paralysis; death [36]. Then, vaginal swabs were taken 5 and 10 days after the assault, and HSV-2 DNA was isolated and analysed by qRT-PCR.

### Statistical Analysis

Data were displayed as the mean ± SEM of three replicates in triplicate, based on at least three independent experiments each with triplicate assays. Data were analysed using the GraphPad Prism software (GraphPad Software Inc., La Jolla, CA, USA). For statistical analysis, differences between treatment groups were compared using Student’s *t*-test and one-way ANOVA test. A *p* value < 0.05 was considered statistically significant.

## Results

### Regulation of Systemic IFN-I Responses by the Commensal Microbiota

**Fig. 1:**
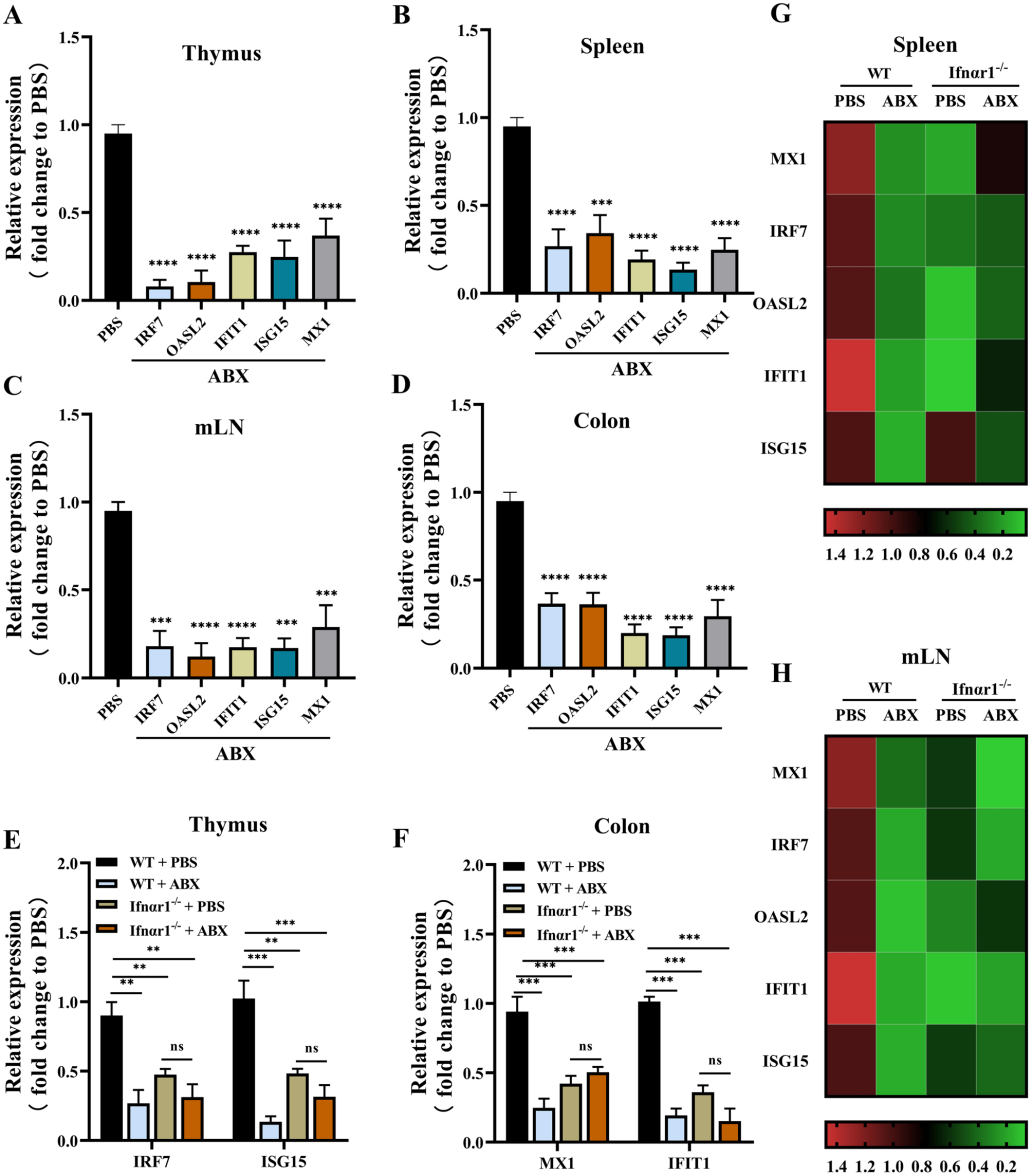
Commensal microbiota regulates host ISGs expression. **A**–**H** RNA was isolated from the thymus, spleen, mLN and colon harvested from WT or Ifnαr1^−/−^ SPF mice with PBS or ABX treatment 10 days post, and ISGs expression was analysed via qRT-PCR. Fold change gene expression in these tissues was calculated compared to PBS-treated WT mice using the 2^−ΔΔCT^ method, with GAPDH as the reference gene. All ISGs expressions of WT mice were depicted in the **A** thymus, **B** spleen, **C** mLN and **D** colon, while Ifnαr1^−/−^ SPF mice were depicted in the **E** *IRF7*, *ISG15* of thymus and **F** *MX1*, *IFIT1* of colon. **G** and **H** Heatmaps of spleen and mLN were showed all the ISGs from WT and Ifnαr1^−/−^ SPF mice. Multiple *t*-test (**E** and **F**) and one-way ANOVA statistical analysis were used and subsequently Tukey’s multiple comparisons test (**A**–**D**) (WT with PBS, *n* = 16; WT with ABX, *n* = 16; Ifnαr1^−/−^ mice with PBS, *n* = 12; Ifnαr1^−/−^ mice with ABX, *n* = 12). *n*, number of mice; ns, not significant; *p < 0.05, **p < 0.01, ***p < 0.001, ****p < 0.0001. **A**–**F** Error bars, ± SEM

To investigate the importance of the commensal microbiota in the response of the host to IFN-I, we first analysed the expression levels of IFN-I-activated antiviral ISGs upon microbiota depletion via treatment with ABX in mice. These commensal microbiota-depleted ABX-treated mice had significantly lower expression levels of ISGs (*IRF7*, *OASL2*, *IFIT1*, *ISG15* and *MX1*) in various systemic tissues such as thymus, spleen, mLN and colon than the PBS-treated WT mice (Fig. 1A–D and G, H). This suggested that commensal microbiota can affect the expression of ISGs in various tissues and organs throughout the body.

As IFN-I is unique in its dynamic functions and in its pattern of expression, to further demonstrate that commensal microbiota can regulate IFN-I expression, the microbiota of Ifnαr1^−/−^ mice was depleted via ABX administration. Relative expression of a panel of ISGs was analysed by qRT-PCR. The expression levels of ISGs in Ifnαr1^−/−^ mice with ABX treatment did not change significantly compared to Ifnαr1^−/−^ mice without ABX treatment (Fig. 1E, F), indicating that depletion of the microbiota does not regulate the response to IFN-I in the absence of IFN-I sensing, and that the microbiota exerts its protective effects via IFN-I. Our results demonstrated that the commensal microbiota can specifically regulate the responses to IFN-I.

### LGG Induces IFN-I Expression

Many commensal microbes colonise the human body [23, 37, 38], but the specific commensal bacteria that affect the responses to IFN-I and the associated specific mechanisms remain unclear. Therefore, we continued to explore the commensal bacteria and the specific mechanisms by which they affect responses to IFN-I. Previous studies dominantly focusing on *Lactobacillus* spp. found that such microbes are vital in the defence of the female genital tract against a wide range of urogenital pathogens. However, the regulatory mechanisms underlying how *lactobacilli* affect viral infections of the female reproductive tract remain unclear. Therefore, we hypothesised that LGG protects against reproductive tract infection by affecting the production of IFN-I. We explored the modulation of host immunity-specific mechanisms.

**Fig. 2:**
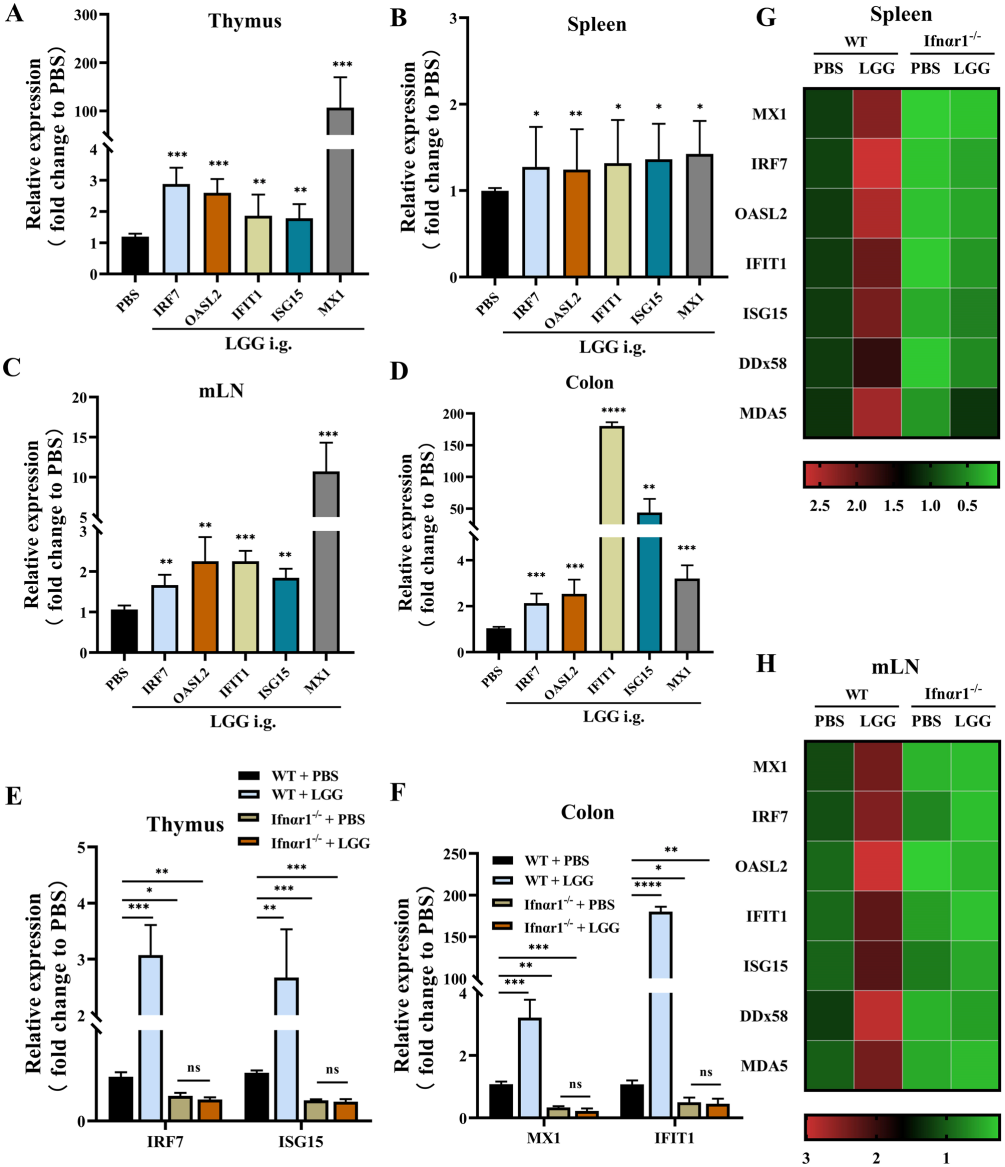
LGG induces IFN-I expression. **A**–**H** RNA was isolated from the spleen, thymus, colon and mLN harvested from WT or Ifnαr1^−/−^ mice with PBS or LGG i.g. 14 days post after ABX treatment, and ISGs expression was analysed via qRT-PCR. Fold change gene expression in these tissues was calculated compared to PBS-treated WT mice using the 2^−ΔΔCT^ method, with GAPDH as the reference gene. All ISGs expressions of WT mice were depicted in the **A** thymus, **B** spleen, **C** mLN and **D** colon, while Ifnαr1^−/−^ SPF mice were depicted in the **E** *IRF7*, *ISG15* of thymus and **F** *MX1*, *IFIT1* of colon. **G** and **H** Heatmaps of spleen and mLN were showed all the ISGs and RIG-I relative genes (*DDx58*, *MDA5*) from WT and Ifnαr1^−/−^ SPF mice. Multiple *t*-test (**E** and **F**) and one-way ANOVA statistical analysis were used and subsequently Tukey’s multiple comparisons test (**A**–**D**). ns, not significant; *p < 0.05, **p < 0.01, ***p < 0.001, ****p < 0.0001. **A**–**F** Error bars, ± SEM

To further verify the LGG ability to enhance IFN-I production, expression of ISGs was analysed by qRT-PCR in ABX-treated mice treated with LGG strains or PBS control. ABX-treated mice administered LGG had significantly higher expression levels of antiviral ISGs (*IRF7*, *OASL2*, *IFIT1*, *ISG15* and *MX1*) in the thymus, spleen, mLN and colon than PBS-treated mice (Fig. 2A–D and G, H). This suggests that LGG can affect the expression of ISGs in different tissues and organs throughout the body.

To further investigate whether LGG can specifically affect IFN-I expression, the microbiota of Ifnαr1^−/−^ mice was modulated via LGG i.g. LGG treatment did not have incremental effect on the expression of ISG in the thymus, spleen, mLN and colon of Ifnαr1^−/−^ mice (Fig. 2E, F and G, H), demonstrating the LGG-specific regulation of the IFN-I response.

### RIG-I Signalling Pathways Affect HSV-2 Infection by Inducing IFN-I Expression

**Fig. 3:**
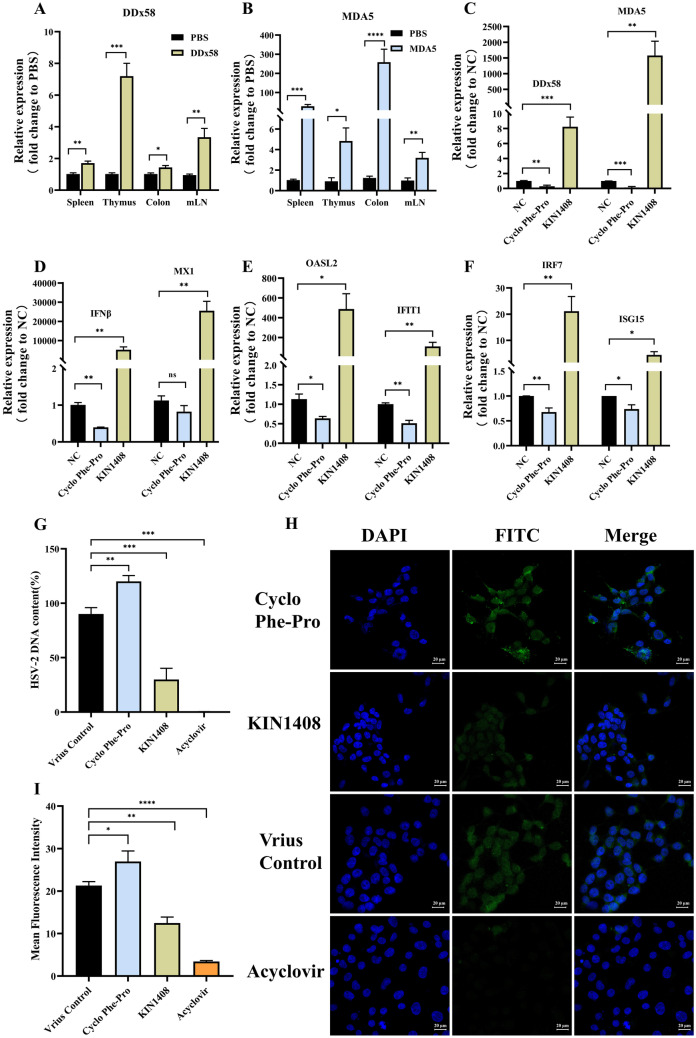
RIG-I signalling pathways affect HSV-2 infection by inducing IFN-I expression. **A**–**B** RNA was isolated from the spleen, thymus, colon and mLN harvested from WT mice with PBS or LGG administration 14 days post after ABX treatment, and RIG-I relative genes (*DDx58*, *MDA5*) expression was analysed via qRT-PCR. Fold change gene expression in these tissues was calculated compared to PBS-treated WT mice using the 2^−ΔΔCT^ method, with GAPDH as the reference gene. **C**–**F** RNA was isolated from HEK293T cells which were harvested after 12 h of co-incubation with RIG-I inhibitor (Cyclo Phe-Pro), RIG-I agonists (KIN1408) and 0.1% DMSO (NC) followed by IFN-β protein stimulation for 2 h, and subsequently were analysed via qRT-PCR. Fold change gene expression in the cells was calculated compared to 0.1% DMSO treatment using the 2^−ΔΔCT^ method, with GAPDH as the reference gene. **G** HSV-2 DNA was isolated from the supernatant of HEK293T cells after 12 h of co-incubation with RIG-I inhibitor (Cyclo Phe-Pro), RIG-I agonists (KIN1408), 0.1% DMSO (control) and acyclovir followed by HSV-2 infection for 18 h. HSV-2 DNA was analysed via qRT-PCR. **H** After incubated with RIG-I inhibitor (Cyclo Phe-Pro), RIG-I agonists (KIN1408), 0.1% DMSO (control) and acyclovir for 12 h, HEK293T cells infected by HSV-2 for 18 h were stained with FITC and DAPI followed by photographed using confocal fluorescence microscopy (bar = 20 μm). Representative images of independent triplicate experiments are shown. **I** The mean fluorescence intensity was analysed by ImageJ. Multiple *t*-test and one-way ANOVA statistical analysis were used and subsequently Tukey’s multiple comparisons test. ns, not significant; *p < 0.05, **p < 0.01, ***p < 0.001. **A**–**G** Error bars, ± SEM

During viral infections, host pattern recognition receptors (PRRs) can recognise the pathogen-associated molecular patterns and induce the production of IFN-I, which exerts a broad-spectrum antiviral effect by binding to the IFN-α/β receptor and inducing the expression of ISGs [16, 39, 40]. RIG-I-like receptors (RLRs) are among the most important intracellular PRRs for innate immunity [41–43]. We analysed the expression profiles of RIG-I, which is a PRR that plays an integral role in the host defence against viruses and can mediate IFN-I-associated immune responses. In the innate immune response, *DDx58* encodes RIG-I, while *MDA5* is an intracellular allosteric RNA monitoring protein and an important member of RLRs family, which recognise cytoplasmic viral nucleic acids and activate downstream cascade signals, leading to the production of IFN-I and pro-inflammatory cytokines in order to resist viral infections, that is the reason why we chose the two genes to analyse [44–46]. Figure 3A, B shows that LGG i.g. to WT mice could significantly increase RIG-I expression (*DDx58*, *MDA5*).

Subsequently, we investigated whether IFN-I expression occurred via RIG-I signalling pathways. HEK293T cells were treated with a RIG-I inhibitor (Cyclo Phe-Pro, a *Vibrio vulnificus* quorum-sensing molecule, inhibits RIG-I polyubiquitination) and RIG-I agonists (KIN1408, an agonist of the RIG-I pathway) to determine whether IFN-I induces ISGs expression and affects virus infection levels by qRT-PCR. Figure 3C shows that the RIG-I inhibitor (Cyclo Phe-Pro) could significantly downregulate the *DDx58* and *MDA5* of RIG-I relative genes while RIG-I agonists (KIN1408) significantly upregulated them. The mRNA expression levels of IFN-β and ISGs, namely, *OASL2*, *IFIT1*, *IRF7* and *ISG15*, in RIG-I signalling pathways were significantly downregulated with RIG-I inhibitor, while upregulation with RIG-I agonists (Fig. 3D–F). Subsequently, the level of viral infection was detected via qRT-PCR and confocal fluorescence microscopy. Figure 3G shows that the inhibition of RIG-I signalling pathways could significantly increase the viral titre in the culture supernatant of HSV-2-infected HEK293T cells, while agonists of RIG-I signalling pathways could significantly decrease the viral titre. The qRT-PCR results were also confirmed via confocal fluorescence microscopy (Fig. 3H-I). The results shown in Fig. 3 demonstrate that the LGG-induced IFN-I response was dependent on RIG-I, affecting HSV-2 infection.

### LGG-Induced IFN-I Enhances Host Anti-HSV-2 Infection

**Fig. 4:**
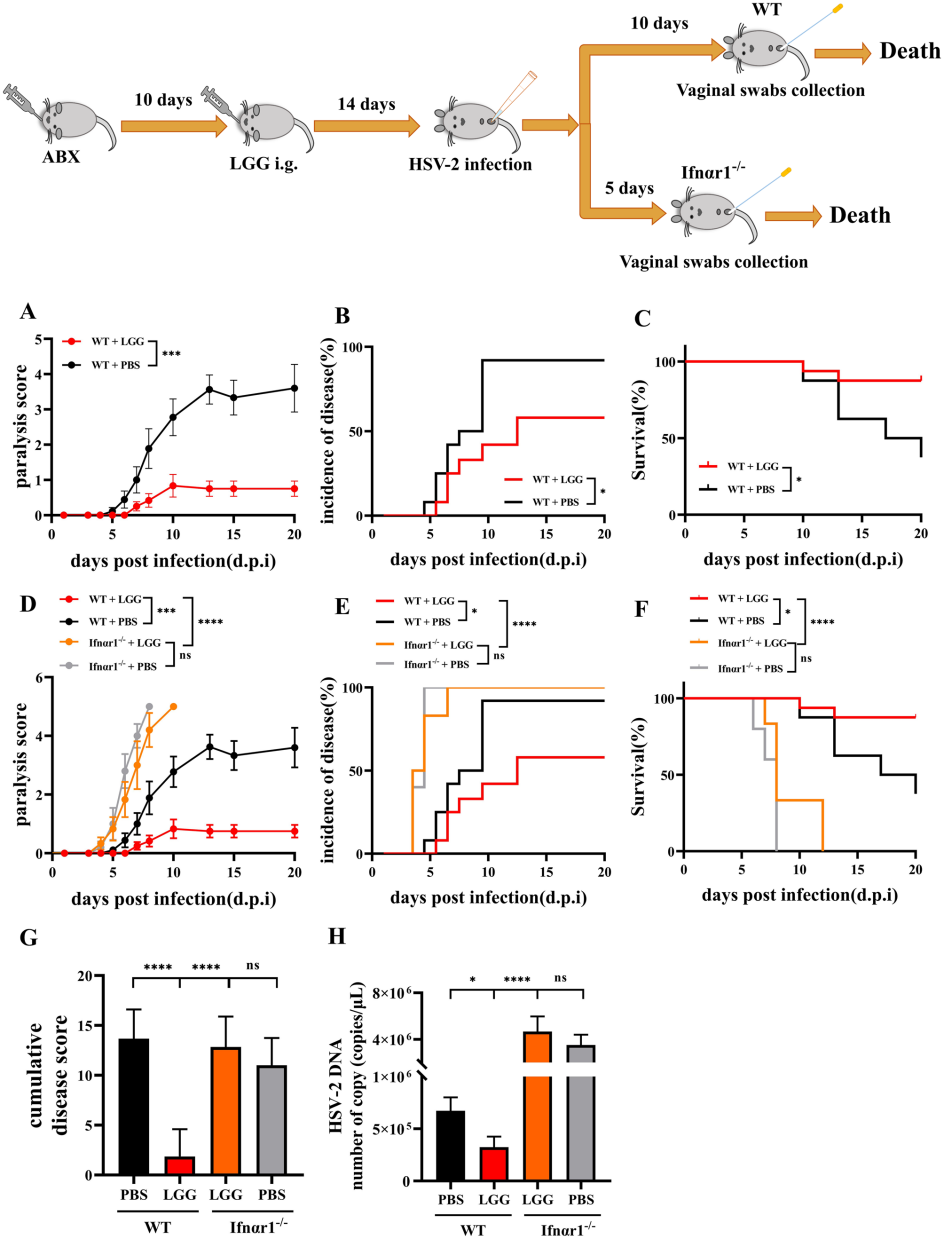
LGG-induced IFN-I enhances host anti-HSV-2 infection. ABX WT or Ifnαr1^−/−^ SPF mice were treated with PBS or LGG for 14 days prior to infection, and 2 mg of sterile PBS suspension medroxyprogesterone acetate was injected subcutaneously into the thighs of mice prior to infection with HSV-2. The attack dose was 1.92 × 10^7^ PFU/mL. The mice were observed on a daily basis for **A** and **D** paralysis score, **B** and **E** percentage of mice with paralysis (disease incidence) and **C** and **F** survival. **G** Sum of daily disease scores per mouse (cumulative disease score) was calculated 20 d.p.i. **H** qRT-PCR analysed the DNA copy number of HSV-2 in WT mice and Ifnar^−/−^ mice. In WT mice vaginal swabs collected on the 10 d.p.i. and Ifnar^−/−^ mice vaginal swabs collected on the 5 d.p.i. Statistical analysis was performed using **A** and **D** linear regression analysis, **B**, **C**, **E** and **F** log-rank test and **G** and **H** one-way ANOVA statistical analysis was used and subsequently Tukey’s multiple comparison test. ns, not significant; *p < 0.05, **p < 0.01, ***p < 0.001, ****p < 0.0001. **A**, **D**, **G** and **H** Error bars, ± SEM

To further assess the anti-viral activity of LGG. WT and Ifnαr1^−/−^ mice were administered LGG every other day via i.g. last for 14 days before challenged with HSV-2. The results showed that the LGG administration significantly alleviated disease severity in WT ABX-treated mice, with lower daily paralysis scores and cumulative disease scores (Fig. 4A, D and G) and disease incidence (Fig. 4B and E), as well as improved survival (Fig. 4C and F). LGG significantly reduced HSV-2 titres in mice vagina (Fig. 4H). HSV-2 titres from the vaginal swabs post infection also confirmed the protective efficacy of LGG against HSV-2 infection in vivo. In addition, the results obtained from observing Ifnαr1^−/−^ mice i.g. administered LGG showed that LGG did not significantly affect susceptibility to HSV-2 infection and the severity of infection in mice (Fig. 4D–H), demonstrating that the antiviral activity of LGG is mediated by IFN-I signalling.

In summary, our findings indicate that the commensal microbiota has a critical role in host immunity to viral infection. Among these microorganisms, LGG could inhibit HSV-2 infection by affecting the expression of IFNs via RIG-I signalling pathways.

## Discussion

Recently, numerous studies have confirmed that the commensal microbiota plays an important role in host immunity against infections, especially viral infections [26, 47]. However, the relationship between the commensal microbiota and viral infections of the female reproductive and genital tract needs to be elucidated. Similar to previous studies [26]. Our study showed that the commensal microbiota can specifically regulate responses to IFN-I expression (Fig. 1). Furthermore, we analysed the specific mechanisms by which the commensal microbiota regulates IFN-I. Previous studies have confirmed that *lactobacilli* play a pivotal role in immunomodulation and viral infections in the host [48]. One previous study focused mainly on *Lactobacillus* spp. showed that it is an important defender of the female genital area against many genitourinary infections, including HIV-1 [49–51]. As a microecological regulator of reproductive vaginal viral infection, LGG is one of the most widely adopted probiotics [33, 52]. Therefore, we investigated whether LGG can regulate IFN-I in hosts and its specific mechanisms. The expression levels of ISGs (*IRF7*, *OASL2*, *IFIT1*, ISG15 and *MX1*) in various systemic tissues throughout the body of the ABX-treated mice were increased significantly after LGG administration (Fig. 2A–D), while Ifnαr1^−/−^mice showed no significant expression increase of these ISGs (Fig. 2E–H).

During viral infections, host PRRs can recognise pathogen-associated molecular patterns and induce the production of IFN-I, which exerts a broad-spectrum antiviral effect by binding to the IFANR1 and inducing the expression of ISGs [39, 40]. The intracellular RLRs are among the most important intracellular PRRs for innate immunity [41–43]. Figures 2H and 3A–B show that LGG administration to ABX-treated mice could significantly increase RIG-I expression.

Subsequently, we investigated whether IFN-I expression occurred via RIG-I signalling pathways. HEK293T cells were treated with a RIG-I inhibitor (Cyclo Phe-Pro) and RIG-I agonists (KIN1408) to determine if IFN-I induces ISGs and virus infection levels by qRT-PCR. Figure 3D–F shows that the mRNA expression levels of IFN-β and ISGs in RIG-I signalling pathways were significantly downregulated with RIG-I inhibitor, while upregulation with RIG-I agonists. Subsequently, the level of viral infection was detected via qRT-PCR and confocal fluorescence microscopy. Figure 3G–I shows that the inhibition of RIG-I signalling pathways could significantly increase the viral titre of HSV-2-infected HEK293T cells, while agonists of RIG-I signalling pathways could significantly decrease the viral titre of HSV-2-infected HEK293T cells. These results demonstrate that the LGG-induced IFN-I response was dependent on RIG-I, affecting HSV-2 infection.

To further evaluate the antiviral activity of LGG, we subsequently determined whether LGG could affect HSV-2 infection via IFN-I. WT or Ifnαr1^−/−^ ABX-treated mice were administered with LGG every other day for 14 days prior to infection with HSV-2. The results showed that WT mice with LGG had significantly alleviated signs of HSV-2 infection and significantly lower viral loads than mice with PBS (Fig. 4A–C and H). In addition, the administration of LGG did not significantly affect the susceptibility to HSV-2 infection and the severity of viral infection in Ifnαr1^−/−^ mice (Fig. 4D–H). These data suggest that LGG cannot alter responses to IFN-I in the absence of IFN-I, demonstrating that the antiviral activity of LGG is mediated through the IFN-I pathway. Therefore, we propose that LGG could inhibit HSV-2 infection by inducing the expression of IFN-I via RIG-I signalling pathways.

There are still a number of potential limitations to the present study. First, although we demonstrate that LGG is mediated by IFN-I signalling and that the IFN-I response depends on RIG-I, we did not analyse the expression of IFN-I and their downstream ISGs after LGG administration to mice in which RIG-I signalling pathways had been inhibited in RIG-knockout mice. Second, this study has not identified the LGG-derived metabolites and metabolic products that affect RIG-I to induce IFN-I production in hosts. These limitations will be addressed in future studies.

## Conclusion

In summary, we show that the level of expression of ISGs through the host IFN-I pathway was downregulated significantly following the depletion of the commensal microbiota due to treatment with ABX. In addition, we confirmed a unique molecular mechanism underlying the induction of IFN-I mediated by the commensal microbiota. In vivo and in vitro, it was confirmed that LGG can suppress HSV-2 infection by inducing IFN-I expression via the RIG-I signalling pathway. Hence, LGG-induced production of IFN-I provides a potential therapeutic approach to combat viral infections.

## Supplementary Information

Below is the link to the electronic supplementary material.

Supplementary Figure 1: Bacterial 16S rDNA in feces of mice with and without ABX treatment were analyzed by qPCR. (TIF 321 KB)

Supplementary Figure 2: ELISA analysis of IFN-β in the colon tissue of WT mice with PBS or LGG i.g. after ABX treatment. Statistical analysis with unpaired t-test. (TIF 668 KB)

## Data Availability

All data generated or analysed during this study are included in this published article.

## Abbreviation

LGG: *Lactobacillus rhamnosus* GG
IFN-I: Interferon type I
ABX: Broad-spectrum antibiotics
HSV-2: Herpes simplex virus type 2
RIG-I: Retinoic acid-inducible gene-I
HIV-1: Human immunodeficiency virus
IFNAR: IFN-I receptor
ISGs: IFN-stimulated genes
SPF: Specific pathogen free
WT: Wild type
Ifnαr1^−/−^: IFNAR1-knockout
mLN: Mesenteric lymphoid node
RNA: Ribonucleic acid
PBS: Phosphate-buffered saline
CFU: Colony-forming units
PFU: Plaque-forming units
IACUC: Institutional Animal Care and Use Committee
qRT-PCR: Quantitative reverse transcriptase‑polymerase chain reaction
cDNA: Complementary DNA
DMSO: Dimethyl sulfoxide
GAPDH: Glyceraldehyde-3-phosphate dehydrogenase
HEK293T: Human embryonic kidney 293 with SV40 T-antigen
BSA: Bovine serum albumin
FITC: Fluorescein isothiocyanate isomer I
DAPI: 4′,6-Diamidino-2′-phenylindole
SEM: Standard error of measurement
IRF7: Interferon regulatory factor 7
OASL2: Oligoadenylate synthetase-like 2
IFIT1: Interferon induced protein with tetratricopeptide repeats 1
ISG15: Interferon-stimulated gene 15
MX1: Myxovirus (influenza virus) resistance 1
DDx58: DEAD (Asp-Glu-Ala-Asp) box polypeptide 58
MDA5: Interferon induced with helicase C domain 1
RLRs: RIG-I-like receptors
i.g.: Intragastric gavage
mRNA: Messenger RNA
PRRs: Pattern recognition receptors
