# Supplementary figures and images for: Lactobacillus rhamnosus GG Regulates Host IFN-I Through the RIG-I Signalling Pathway to Inhibit Herpes Simplex Virus Type 2 Infection
Source: Probiotics Antimicrob Proteins. 2023 Aug 25;16(6):1966–78. doi: 10.1007/s12602-023-10137-8 (PMC11573810; doi:10.1007/s12602-023-10137-8)

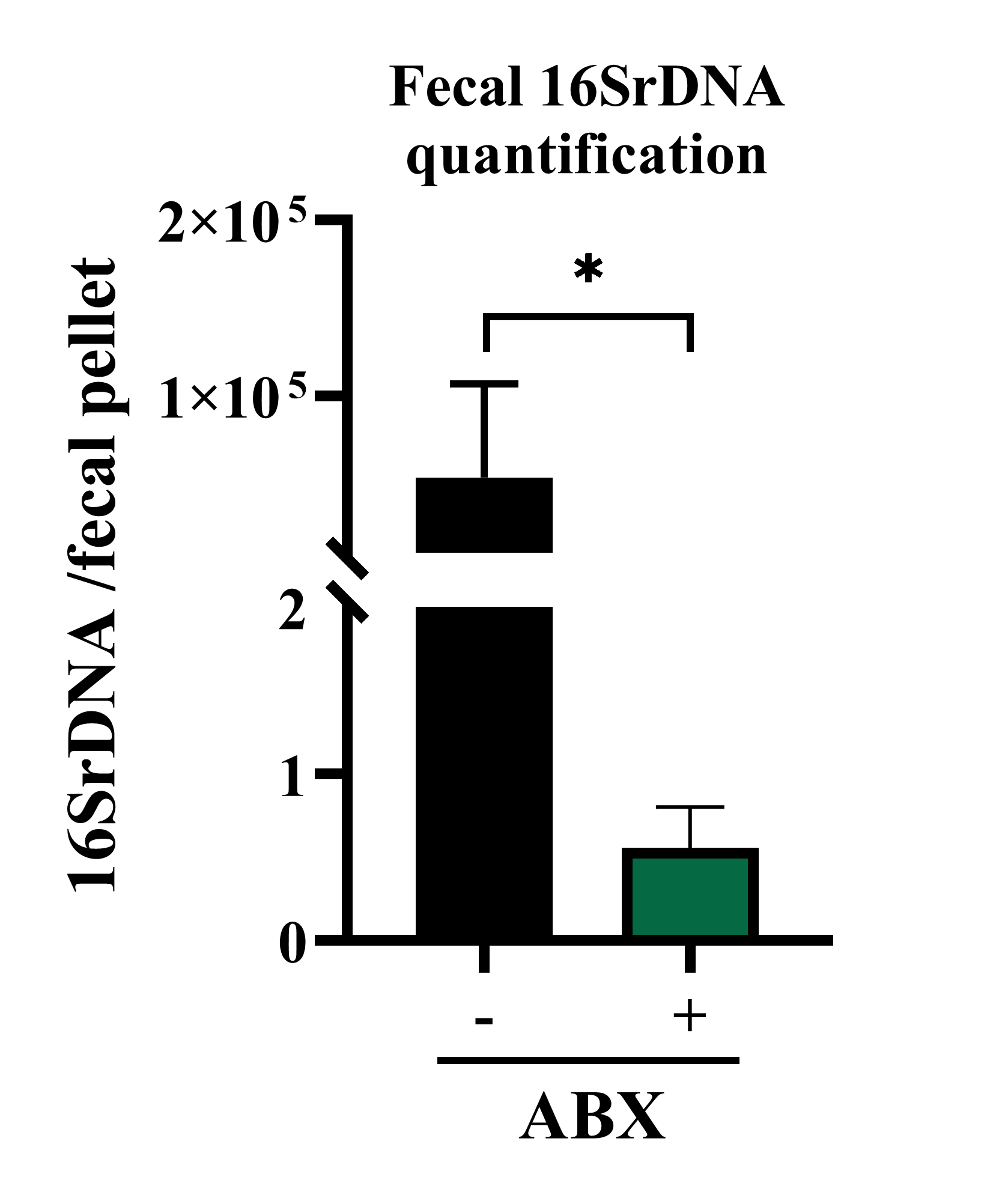

Supplement: Supplementary file 1 — Supplementary Figure 1: Bacterial 16S rDNA in feces of mice with and without ABX treatment were analyzed by qPCR. (TIF 321 KB) [file 12602_2023_10137_MOESM1_ESM.tif]

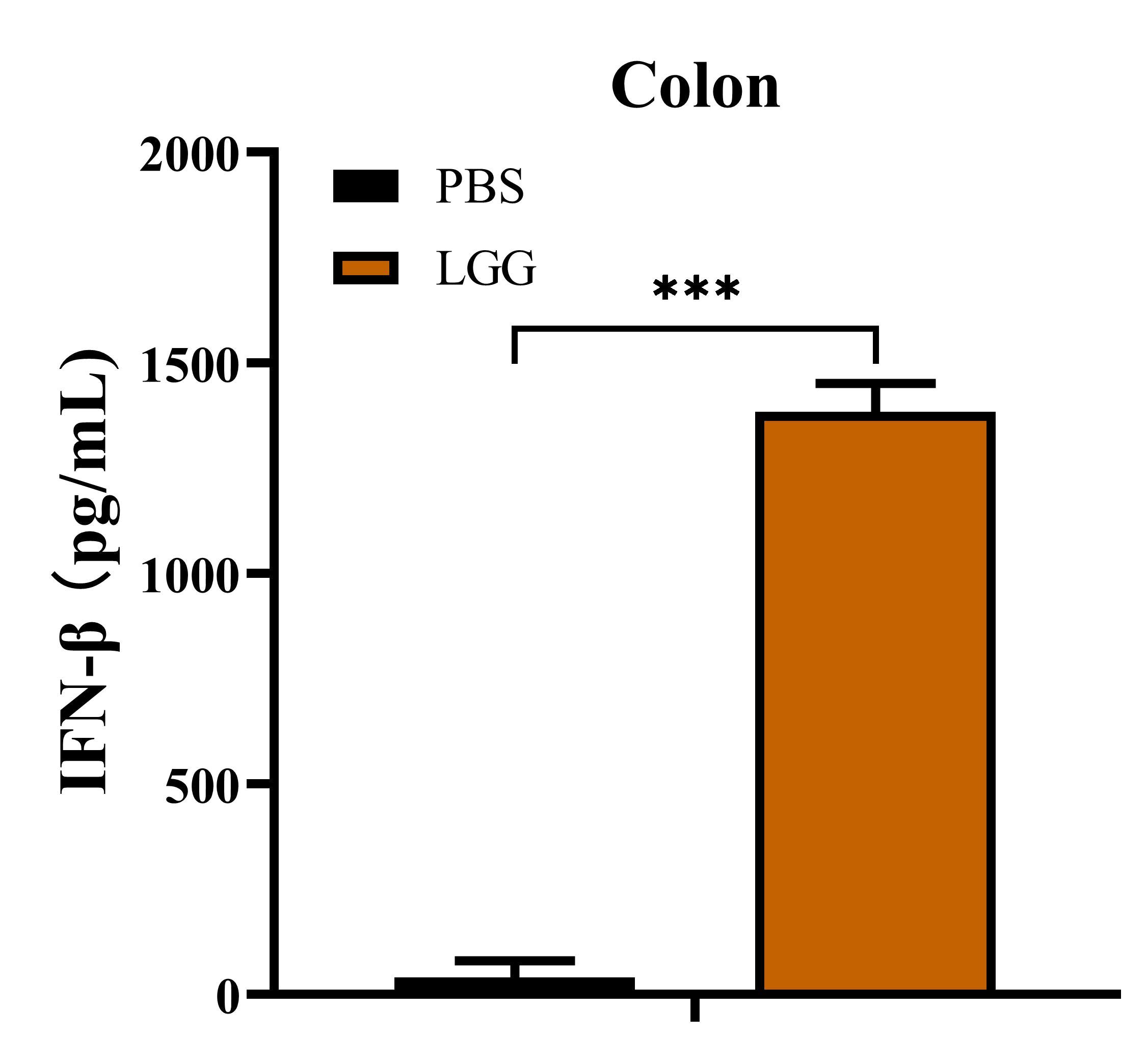

Supplement: Supplementary file 2 — Supplementary Figure 2: ELISA analysis of IFN-β in the colon tissue of WT mice with PBS or LGG i.g. after ABX treatment. Statistical analysis with unpaired t-test. (TIF 668 KB) [file 12602_2023_10137_MOESM2_ESM.tif]
